# Thermophysical Study of 1-Butyl-2-methylpyridinium bis(trifluoromethylsulfonyl)imide and 1-Butyl-4-methylpyridinium bis(trifluoromethylsulfonyl)imide

**DOI:** 10.1021/acs.jced.4c00255

**Published:** 2024-09-10

**Authors:** Hamid Djebouri, Saeda Didaoui, Sahar Mrad, Alberto Lafuente, Carlos Lafuente

**Affiliations:** † Laboratory of Thermodynamics and Molecular Modeling, Faculty of Chemistry, University of Sciences and Technology Houari Boumediene, BP 32, El Alia, Algiers 16111, Algeria; ‡ Departamento de Química Física, Facultad de Ciencias, Universidad de Zaragoza, 50009 Zaragoza, Spain; § Laboratoire des Matériaux, Cristallochimie et Thermodynamique Appliquée, LR15ES01, Département de Chimie, Université de Tunis El Manar, Faculté des Sciences, 2092 Tunis, Tunisia

## Abstract

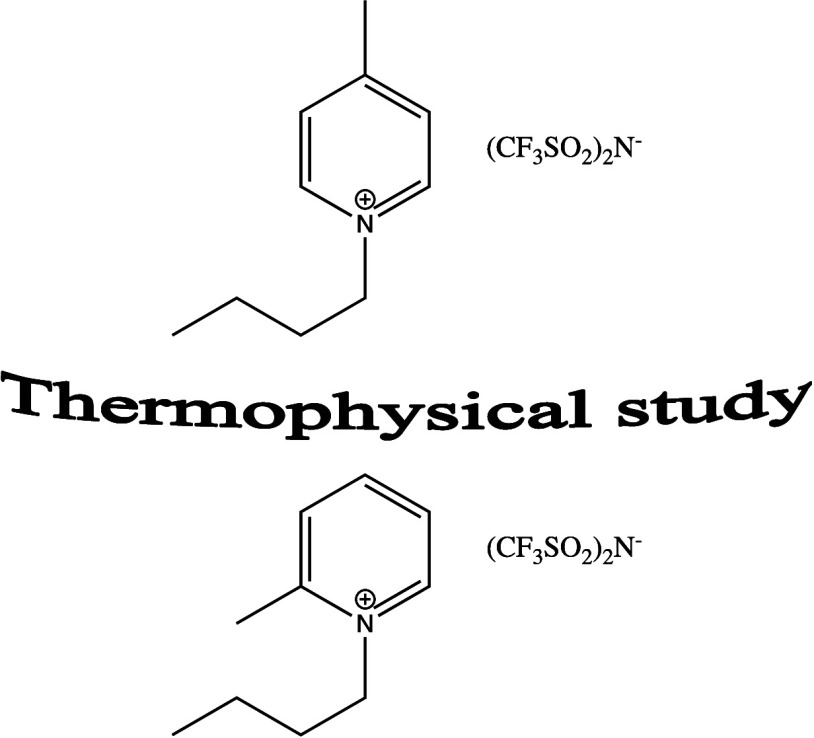

Ionic liquids are of great interest for scientists and chemical industries owing to their fascinating properties and their versatile applications. Herein, we focus on the thermophysical properties of two isomeric pyridinium-based ionic liquids: 1-butyl-2-methylpyridinium bis(trifluoromethylsulfonyl)imide and 1-butyl-4-methylpyridinium bis(trifluoromethylsulfonyl)imide. Experimental investigation of the liquids involved the measurement of their thermodynamic and transport properties, namely, density, speed of sound, refractive index, surface tension, isobaric molar heat capacity, kinematic viscosity, and electrical conductivity. These measurements were performed in the temperature range of 278.15–323.15 K and ambient pressure of 100 kPa. Subsequently, additional properties such as isobaric expansivity, isentropic compressibility, free molar volume, and dynamic viscosity were calculated. Results were discussed considering the structural differences between the isomers, particularly focusing on the influence of the alkyl chain position in the cation on the investigated properties.

## 1 Introduction

In recent years, ionic liquids (ILs) have become one of the most interesting and rapidly developing areas of research. This success primarily arises from their transformation into new ecological solvents owing to their safety, sustainability, and nonvolatility. In addition, they are nonflammable and can be easily recyclable. Second, ILs are distinguished by their unique properties such as negligible vapor pressure under ambient conditions and long-range thermal stability. Moreover, they exhibit a strong ability to solvate organic and inorganic compounds. Thus, they are considered promising alternatives to volatile organic solvents.^1−3^

Furthermore, as design solvents, ILs can be tailored by varying combinations of an anion or a cation to adapt to reaction conditions and achieve the desired properties. The large variety of possible combinations expands the number of ILs under study and their application in various fields of research and industry such as synthesis,^1,4−6^ catalysis,^1,6−9^ biology,^3,6^ electrochemistry,^1,6,10^ analytics,^3,6^ engineering,^1,3,6^ and liquid–liquid extraction.^11^ Additionally, they are recognized as good conductors with values reaching up to 20 mS/cm, making them suitable electrolytes.^10,12^

Prominent ILs comprise bulky organic cations characterized by delocalized charge, namely, imidazolium, pyridinium, ammonium, and phosphonium cations, paired with either organic or inorganic anions such as tetrafluoroborate, ethanoate, nitrate, or bis(trifluoromethylsulfonyl)imide.^10^ Particularly, pyridinium-based ILs have demonstrated potential in biological activities and therapeutic applications.^3^ Alternatively, hydrophobic ILs containing specific anions such as bis(trifluoromethylsulfonyl)imide conduct to immiscible mixtures when mixed with an excess of water, and therefore, these types of mixtures are of great relevance in future liquid extraction processes.^13^ To enhance our understanding of these ILs, we studied the thermophysical properties of two isomeric pyridinium-based ILs combined with the bis(trifluoromethylsulfonyl)imide anion. Furthermore, knowledge regarding the thermophysical properties of pure ILs can provide information about intermolecular interactions in the fluid and can improve the development and refining of thermodynamic models, thereby helping the optimization of the future applications of ILs.^2^

Herein, we examine two pyridinium-based ILs, namely, 1-butyl-2-methylpyridinium bis(trifluoromethylsulfonyl)imide ([b2mpy][Tf_2_N]) and 1-butyl-4-methylpyridinium bis(trifluoromethylsulfonyl)imide ([b4mpy][Tf_2_N]). Further, the impact of the position of the alkyl substituent in the cation on the thermophysical properties of the liquids is explored. This study also measures the various properties including density, speed of sound, refractive index, surface tension, isobaric molar heat capacity, kinematic viscosity, and electrical conductivity in the temperature range of 278.15–338.15 K with a temperature increment of 2.5 K, all at ambient pressure (100 kPa). Using the experimental data, we also determine the isobaric expansivity, isentropic compressibility, free volume, and dynamic viscosity. Additionally, the entropies and enthalpies of surface formation per unit surface area are calculated.

A survey of the literature revealed several studies reporting the thermodynamic and transport properties of these ILs. Heat capacities,^14,15^ densities, dynamic viscosities, surface tensions,^16^ and solubility in water^13^ were experimentally determined for both ILs. Most of the data reported concerned [b4mpy][Tf_2_N]. Studies included density, dynamic viscosity, refractive index, thermal decomposition, and electrical conductivity.^17−21^ Andanson^22^ quantified the effect of the presence of water as an impurity on the physicochemical properties of [b4mpy][Tf_2_N]. Papaiconomou^23^ reported the synthesis of [b4mpy][Tf_2_N] and investigated its thermal stability, density, and solubility in water. Regarding [b2mpy][Tf_2_N], there are few studies available. Therefore, the findings of this study contributes to a better understanding of the behavior of these liquids, specifically focusing on the effect of the methyl position in the pyridinium ring on the thermophysical properties.

## 2 Experimental Section

The ILs used in this study and their corresponding information are summarized in Table 1. Because the presence of water considerably impacts the thermophysical properties of ILs, it is crucial to minimize it as much as possible in the studied ILs. Therefore, before measurement, the ILs were dried for 24 h under a vacuum pressure of ∼0.05 kPa. After drying, the water content was analyzed via Karl Fischer titration using a Crison KF 1S-2B automatic titrator and reassessed after experimental measurement with no significant increase observed. The measurements were performed at ambient pressure (100 kPa) and temperatures of 278.15–328.15 K.

**Table 1:** Ionic Liquids

chemical name	CAS number	source	purity^a^,^b^ (mass fraction)	water content (mass fraction)	halides content (mass fraction)
1-butyl-2-methylpyridinium bis(trifluoromethylsulfonyl)imide	384347-09-5	iolitec	0.99	0.00004	<0.0001
1-butyl-4-methylpyridinium bis(trifluoromethylsulfonyl)imide	475681-62-0	iolitec	0.99	0.00005	<0.0001

a As stated by the supplier by NMR analysis.

b Ionic liquids were dried under vacuum of 0.05 kPa for 24 h.

Density (ρ) and speed of sound (*c*) were measured using an Anton Paar DSA 5000 vibrating tube densimeter and sound analyzer, automatically thermostated, respectively. Before measurements, instrument calibration was performed using ultrapure water and dry air, according to the manufacturer’s instructions. The density of the sample was determined by measuring the oscillation period of the U-shaped tube, with the viscosity errors in density automatically corrected using the densimeter. The speed of sound was determined using an acoustic time-of-flight method^24^ operating at a frequency of 3 MHz. The combined expanded uncertainties for density and speed of sound were estimated to be 10^–3^ g·cm^–3^ and 1 m·s^–1^, respectively, and the standard uncertainty for temperature was 0.01 K.

Refractive index (*n*_D_) was measured using an Abbemat-HP Dr Kernchen automatic refractometer operating at a wavelength λ of 589.3 nm (sodium D). The apparatus was calibrated with distilled deionized water. The temperature of the sample and the internal temperature of the optical system were maintained within ±0.01 K owing to the two built-in Peltier devices. The combined expanded uncertainty for refractive index was estimated to be 10^–4^.

Surface tension (σ) was obtained using the drop volume technique via a Lauda TVT-2 tensiometer.^25^ The temperature was controlled using a Lauda E-200 thermostat. The standard uncertainty for temperature was 0.1 K, and the combined expanded uncertainty for surface tension was 1 mN·m^–1^.

Isobaric molar heat capacity (*C*_p,m_) was determined through differential scanning calorimetry using a TA Instruments DSC Q2000 calorimeter at a heating rate of 20 K/min.^26^ A synthetic sapphire disk provided by TA Instruments served as the reference standard. The standard uncertainty for temperature was 0.01 K, and the combined expanded uncertainty for isobaric heat capacity was estimated to be 10 J·mol^–1^ K^–1^.

Kinematic viscosity (ν) was measured using an Ubbelohde viscometer accompanied by a Schott–Gerate automatic measuring unit model AVS-440 using various capillaries. These capillaries were originally calibrated, and this calibration was assessed with different liquids. The flow time *t* of the sample was determined with at least three measurements taken for each data. A Schott–Gerate thermostat model CT 1150/2 was used to maintain the sample temperature constant within ±0.01 K. The combined expanded uncertainty for kinematic viscosity was estimated to be 5 mm·s^–1^. From the experimental density and kinematic viscosity values, the dynamic viscosity can be calculated using the formula η = ρ ν.

Electrical conductivity (κ) was obtained using a Crison conductimeter model LPG31 working at an alternating frequency of 2 kHz. The calibration of the cell was conducted using two aqueous solutions of KCl with different concentrations. To enhance precision, the measurements were repeated thrice and then averaged. The sample temperature was kept constant using a Lauda E-200 thermostat. The standard uncertainty for temperature was 0.01 K, and the combined expanded uncertainty for electrical conductivity was 0.20 mS·cm^–1^. Further details of the experimental procedure can be found in a previous paper.^26^

## 3 Results and Discussion

**Figure 1:**
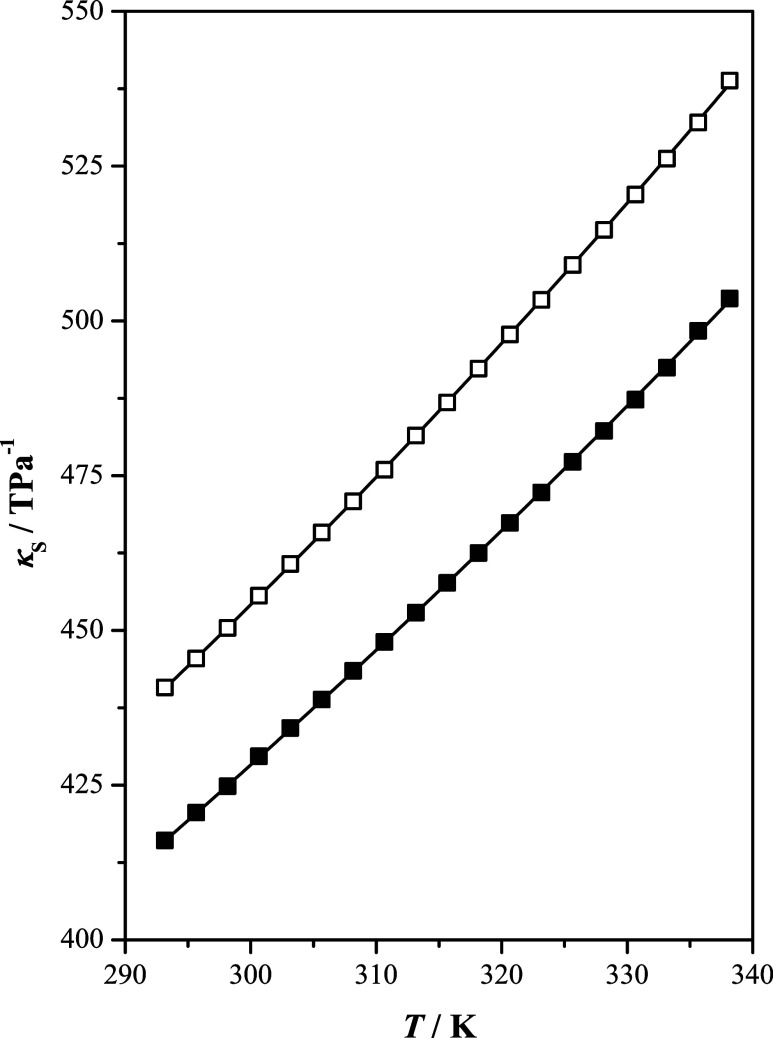
Isentropic compressibility, κ_S_, as a function of temperature, *T*, at *p* = 100 kPa of the studied ionic liquids: [b2mpy][Tf_2_N] (■); [b4mpy][Tf_2_N] (□); (—) correlated values.

**Figure 2:**
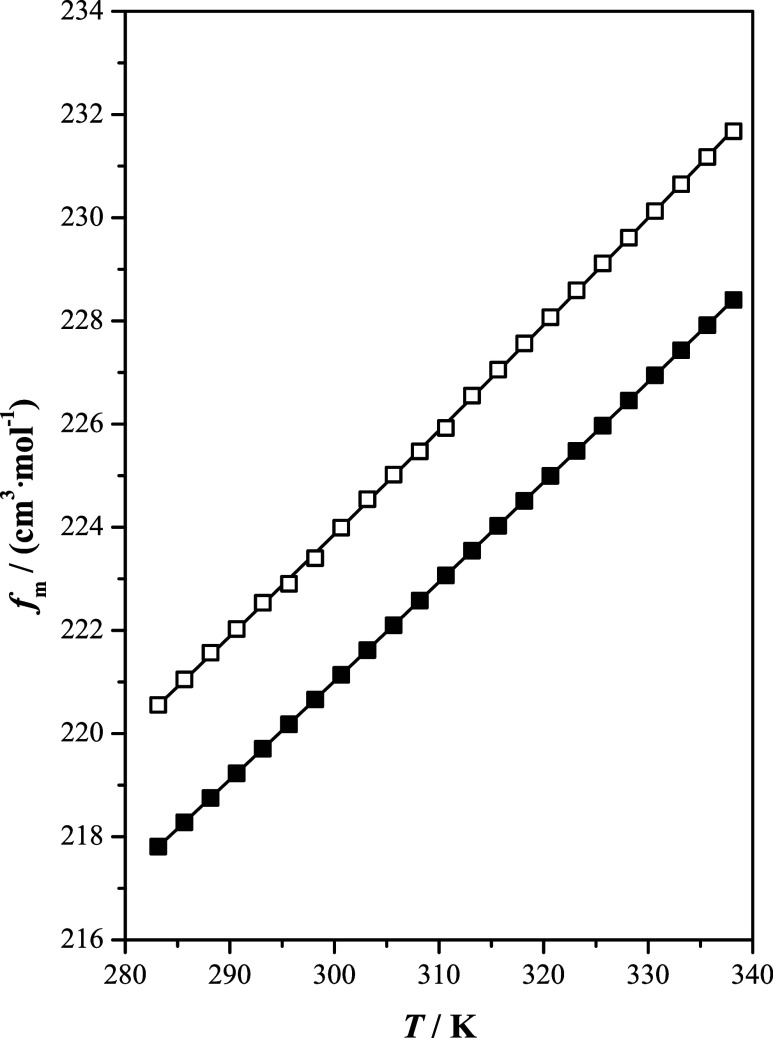
Free volume, *f*_m_, as a function of temperature, *T*, at *p* = 100 kPa of the studied ionic liquids: [b2mpy][Tf_2_N] (■); [b4mpy][Tf_2_N] (□); (—) correlated values.

**Figure 3:**
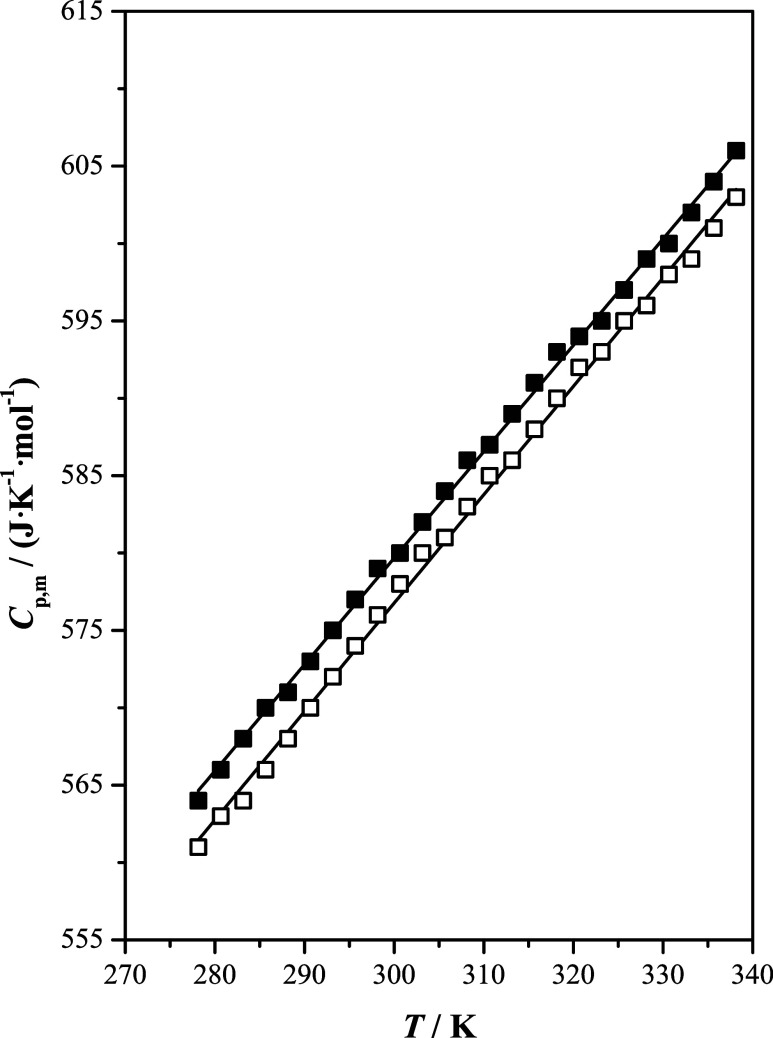
Isobaric molar heat capacity, *C*_p,m_, as a function of temperature, *T*, at *p* = 100 kPa of the studied ionic liquids: [b2mpy][Tf_2_N] (■); [b4mpy][Tf_2_N] (□); (—) correlated values.

**Figure 4:**
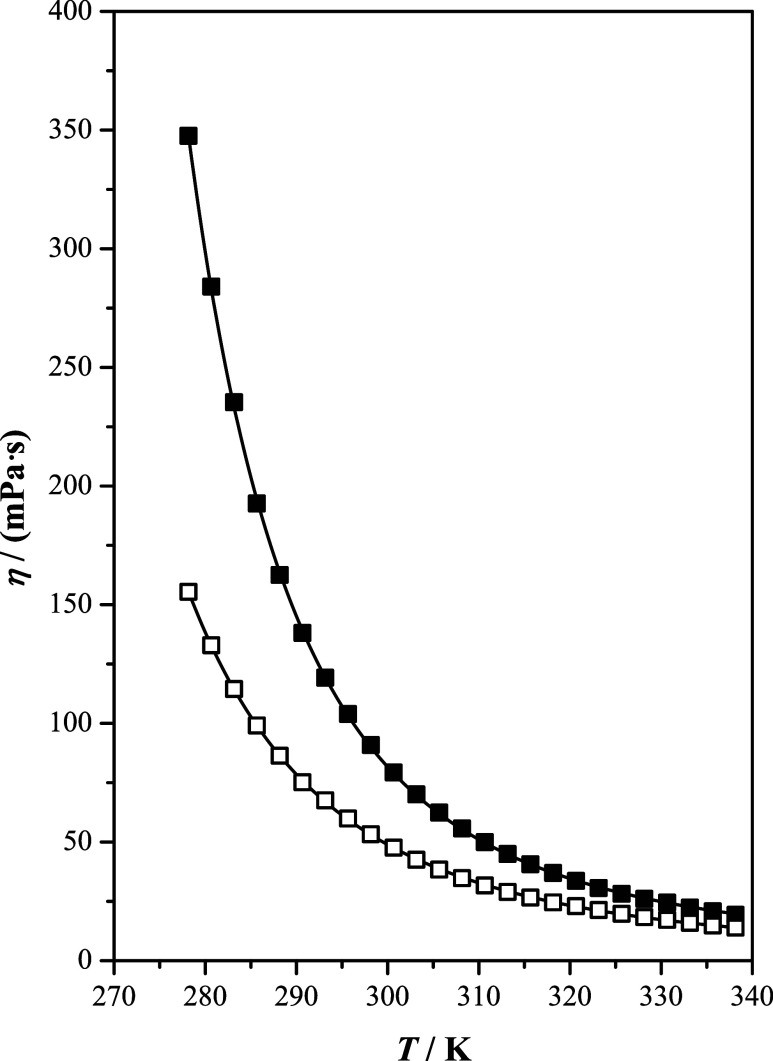
Dynamic viscosity, η, as a function of temperature, *T*, at *p* = 100 kPa of the studied ionic liquids: [b2mpy][Tf_2_N] (■); [b4mpy][Tf_2_N] (□); (—) correlated values.

**Figure 5:**
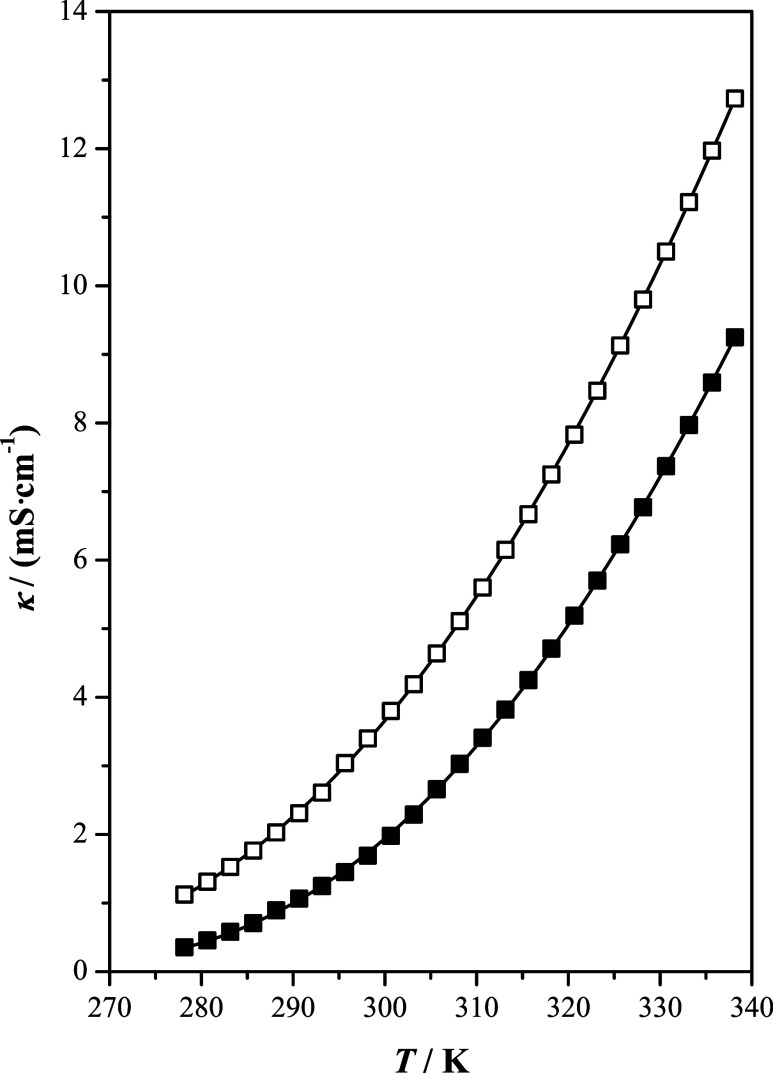
Electrical conductivity, κ, as a function of temperature, *T*, at *p* = 100 kPa of the studied ionic liquids: [b2mpy][Tf_2_N] (■); [b4mpy][Tf_2_N] (□); (—) correlated values.

Table 2 presents the thermophysical properties of the two ILs measured in this study along with some derived properties. The plots for these properties as a function of temperature are shown in Figures 1–5 and in the associated content, Figures S.1-S.4.

**Table 2:** Experimental Thermophysical Properties and Some Derived Properties of the Ionic Liquids as a Function of Temperature, *T*, and at Ambient Pressure, *p* = 100 kPa: Density, ρ, Speed of Sound, *c*, Isentropic Compressibility, κ_S_, Refractive Index, *n*_D_, Free Volume, *f*_m_, Surface Tension, σ, Isobaric Molar Heat Capacity, *C*_p,m_, Kinematic Viscosity, ν, Dynamic Viscosity, η, and Electrical Conductivity, κ^a^

*T* (K)	ρ (g·cm^–3^)	*c* (m·s^–1^)	κ_S_ (TPa^–1^)	*n*_D_	*f*_m_ (cm^3^·mol^–1^)	σ (mN·m^–1^)	*C*_p.m_ (J·K^–1^·mol^–1^)	ν (mm·s^–1^)	η (mPa·s)	κ (mS·cm^–1^)
1-Butyl-2-methylpyridinium bis(trifluoromethylsulfonyl)imide										
278.15	1.4455					35.1	564	240.4	347.6	0.355
280.65	1.4431					35.0	566	196.8	284.1	0.455
283.15	1.4408			1.45407	217.810	34.9	568	163.3	235.3	0.581
285.65	1.4385			1.45331	218.281	34.8	570	134.0	192.7	0.709
288.15	1.4361			1.45258	218.752	34.7	571	113.2	162.5	0.894
290.65	1.4338			1.45180	219.230	34.5	573	96.33	138.1	1.064
293.15	1.4314	1295.8	416.1	1.45103	219.707	34.4	575	83.31	119.2	1.249
295.65	1.4291	1289.9	420.6	1.45027	220.181	34.3	577	72.72	103.9	1.452
298.15	1.4268	1284.4	424.8	1.44952	220.662	34.2	579	63.72	90.91	1.692
300.65	1.4244	1278.2	429.7	1.44877	221.139	34.0	580	55.70	79.35	1.981
303.15	1.4221	1272.5	434.2	1.44801	221.619	33.9	582	49.31	70.12	2.29
305.65	1.4198	1266.9	438.8	1.44725	222.099	33.8	584	43.96	62.42	2.66
308.15	1.4175	1261.3	443.5	1.44649	222.580	33.7	586	39.36	55.78	3.03
310.65	1.4152	1255.7	448.2	1.44574	223.065	33.5	587	35.34	50.01	3.41
313.15	1.4129	1250.1	452.9	1.44497	223.546	33.4	589	31.85	45.00	3.82
315.65	1.4106	1244.5	457.7	1.44422	224.027	33.4	591	28.85	40.67	4.25
318.15	1.4083	1239.1	462.5	1.44345	224.512	33.1	593	26.25	36.97	4.71
320.65	1.4060	1233.6	467.4	1.44270	224.996	33.0	594	23.94	33.66	5.19
323.15	1.4038	1228.2	472.3	1.44194	225.482	32.9	595	21.85	30.67	5.70
325.65	1.4015	1222.7	477.2	1.44119	225.968	32.7	597	20.13	28.22	6.23
328.15	1.3992	1217.4	482.2	1.44042	226.457	32.6	599	18.67	26.12	6.77
330.65	1.3969	1212.0	487.3	1.43968	226.945	32.5	600	17.55	24.52	7.37
333.15	1.3947	1206.7	492.4	1.43894	227.431	32.4	602	16.09	22.44	7.97
335.65	1.3924	1200.4	498.4	1.43821	227.918	32.2	604	14.99	20.88	8.59
338.15	1.3902	1195.1	503.6	1.43747	228.407	32.1	606	13.94	19.38	9.25
1-Butyl-4-methylpyridinium bis(trifluoromethylsulfonyl)imide										
278.15	1.4307					34.1	561	108.7	155.5	1.124
280.65	1.4283					34.1	563	93.08	132.9	1.311
283.15	1.4260			1.45098	220.555	33.9	564	80.30	114.5	1.527
285.65	1.4236			1.45020	221.051	33.8	566	69.64	99.14	1.766
288.15	1.4210			1.44942	221.569	33.7	568	60.79	86.38	2.03
290.65	1.4188			1.44865	222.028	33.6	570	53.00	75.20	2.31
293.15	1.4164	1265.6	440.8	1.44788	222.537	33.4	572	47.74	67.63	2.61
295.65	1.4148	1259.6	445.5	1.44710	222.905	33.4	574	42.36	59.93	3.04
298.15	1.4125	1253.7	450.4	1.44633	223.400	33.2	576	37.75	53.33	3.40
300.65	1.4095	1247.9	455.6	1.44556	223.991	33.1	578	33.83	47.68	3.80
303.15	1.4067	1242.0	460.7	1.44464	224.543	33.0	580	30.26	42.57	4.19
305.65	1.4046	1236.3	465.8	1.44401	225.018	32.9	581	27.35	38.42	4.64
308.15	1.4025	1230.5	470.9	1.44323	225.471	32.7	583	24.81	34.79	5.11
310.65	1.4005	1224.8	476.0	1.44246	225.924	32.7	585	22.68	31.77	5.60
313.15	1.3974	1219.1	481.5	1.44169	226.551	32.5	586	20.75	29.00	6.15
315.65	1.3950	1213.4	486.8	1.44091	227.055	32.4	588	19.09	26.63	6.67
318.15	1.3927	1207.7	492.3	1.44014	227.563	32.3	590	17.66	24.60	7.25
320.65	1.3903	1202.0	497.8	1.43936	228.070	32.2	592	16.55	23.00	7.83
323.15	1.3880	1196.3	503.4	1.43853	228.594	32.1	593	15.41	21.38	8.47
325.65	1.3856	1190.7	509.0	1.43772	229.115	31.9	595	14.29	19.80	9.13
328.15	1.3833	1185.1	514.7	1.43705	229.613	31.8	596	13.28	18.38	9.80
330.65	1.3809	1179.6	520.4	1.43629	230.129	31.7	598	12.47	17.23	10.50
333.15	1.3786	1174.1	526.2	1.43552	230.649	31.6	599	11.62	16.01	11.22
335.65	1.3761	1168.7	532.0	1.43478	231.176	31.5	601	10.82	14.89	11.97
338.15	1.3739	1162.2	538.8	1.43403	231.676	31.3	603	10.17	13.98	12.73

a Standard uncertainties *u* are *u*(*T*) = 0.1 K for surface tension, and *u*(*T*) = 0.01 K for the rest of properties, *u*(*p*) = 1 kPa, and the combined expanded uncertainties *U*_c_ are *U*_c_(ρ) = 10^–3^ g·cm^–3^, *U*_c_(*c*) = 1 m·s^–1^, *U*_c_(*n*_D_) = 5 × 10^–5^, *U*_c_(σ) = 1 mN·m^–1^, *U*_c_(*C*_p,m_) = 10 J·K^–1^·mol^–1^, *U*_c_(ν) = 5 mm·s^–1^, *U*_c_(κ) = 0.20 mS·cm^–1^, with 0.95 level of confidence (*k* = 2).

The majority of the studied thermophysical properties showed a linear relationship with temperature. Therefore, these properties were correlated using the following equation
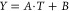
1where *Y* is the studied property and *A* and *B* are adjustable parameters. The best linear fitting parameters along with the absolute average relative deviation (AARD) are gathered in Table 3. AARD is defined as follows
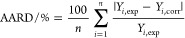
2

**Table 3:** Fitting Parameters along with the Absolute Average Relative Deviations, AARD, for the Measured Properties

property	*A*	*B*	*C*	AARD (%)
1-Butyl-2-methylpyridinium bis(trifluoromethylsulfonyl)imide				
ρ (g·cm^–3^)	–0.00092	1.7018		0.01
*c* (m·s^–1^)	–2.260	1958.6		0.05
*n*_D_	–0.000303	1.53974		0.01
σ (mN·m^–1^)	–0.0507	49.3		0.00
*C*_p,m_ (J·mol·K^–1^)	0.688	373		0.07
η^a^ (mPa·s)	0.503	501.6	201.4	0.90
κ^b^ (mS·cm^–1^)	318.8	–441.4	213.5	0.94
1-Butyl-4-methylpyridinium bis(trifluoromethylsulfonyl)imide				
ρ (g·cm^–3^)	–0.00095	1.6950		0.02
*c* (m·s^–1^)	–2.312	1943.5		0.04
*n*_D_	–0.000309	1.53846		0.02
σ (mN·m^–1^)	–0.0467	47.1		0.04
*C*_p,m_ (J·mol·K^–1^)	0.701	369		0.43
η (mPa·s)	0.273	621.7	180.2	0.62
κ (mS·cm^–1^)	483.0	–543.6	188.7	0.43

a *A* = η_0_; *C* = *T*_0_.

b *A* = κ_0_; *C* = *T*_0_.

The density dependence of temperature for the investigated ILs is illustrated in Figure S.1. As temperature increases, the density values for both ILs decrease. This can be explained by the thermal expansion of the IL and the weakening of electrostatic forces between ions with increasing temperature.^28,29^ Because both ILs possess the same anion, the density values were higher for [b2mpy][Tf_2_N] than [b4mpy][Tf_2_N] in the entire range of temperatures (from 278.15 to 338.15 K). According to Bittner et al.,^16^ the introduction of the methyl group in the *ortho* position of the pyridine ring contributes to better structural fitting or greater intermolecular interactions, resulting in higher density. Isobaric expansivity (α_p_) can be obtained from the dependence of molar volume on temperature at constant pressure. The isobaric expansivity values at *T* = 298.15 K of the ILs [b2mpy][Tf_2_N] and [b4mpy][Tf_2_N] were 0.655 and 0.682 kK^–1^, respectively. Again, the position of the methyl group influences the behavior of the isobaric expansivity being higher for [b4mpy][Tf_2_N] than [b2mpy][Tf_2_N].

Isentropic compressibility was calculated from the density and speed of sound experimental values using the Newton–Laplace equation, κ_*S*_ = 1/(ρ *c*^2^), assuming that ultrasonic absorption is negligible. The determined isentropic compressibility (κ_S_) for the studied ILs as a function of temperature is shown in Figure 1. The isentropic compressibility for ILs increases with increasing temperature. The isentropic compressibilities of [b2mpy][Tf_2_N] and [b4mpy][Tf_2_N] depend on the substituent position in the cation. Smaller values were observed for [b2mpy][Tf_2_N] than for [b4mpy][Tf_2_N] in the entire temperature range. Because isentropic compressibility is related to the packing arrangement,^28^ it is expected that IL with a more compact structure will tend to have lower isentropic compressibility.

Free volume (*f*_m_) can be obtained from molar volume and molar refraction via the Lorentz–Lorenz relation:
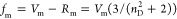
3A similar trend was observed for the free volume, which provides an indication of the unoccupied volume by ions in the fluid. Figure 2 illustrates that this property increases with temperature and that larger values were obtained for [b4mpy][Tf_2_N] than for [b2mpy][Tf_2_N]. Similarly, similar to isentropic compressibility, ions with more compact arrangement in the fluid will exhibit smaller free volume values.

In Figure S.4, the surface tensions of [b2mpy][Tf_2_N] and [b4mpy][Tf_2_N] are plotted as a function of temperature. As temperature rises, surface tension values decrease. Particularly, the surface tension of [b2mpy][Tf_2_N] is higher than that of its isomer [b4mpy][Tf_2_N] and the difference between the values decreases with increasing temperature. In general, the cohesive forces occurring in the bulk and the orientation of ions at the surface play a significant role in affecting this property.^27,30^ In fact, surface tension increases with these interactions in the liquid, while it diminishes with packing efficiency at the surface.^31^ Based on this, we can conclude that [b2mpy][Tf_2_N] exhibits stronger interactions in the bulk while [b4mpy][Tf_2_N] has a higher level of organization at the surface. The study of surface phenomena can be further emphasized through experimental surface tension measurements by examining the entropy (*S*^σ^) and the enthalpy of surface formation (*H*^σ^).^32^ These surface thermodynamic properties were calculated using the quasilinear dependence of the surface tension with temperature. The entropies of the surface formation values of the ILs [b2mpy][Tf_2_N] and [b4mpy][Tf_2_N] were 0.0507 and 0.0467 mN·m^–1^·K^–1^, respectively. In agreement with previously reported conclusions, a lower surface entropy suggests a more organized liquid surface structure for [b4mpy][Tf_2_N].^16,33^ The enthalpies of surface formation values were 49.3 and 47.1 mN·m^–1^ for [b2mpy][Tf_2_N] and [b4mpy][Tf_2_N] at *T* = 298.15 K, respectively. On comparing these values, the enthalpy of surface formation was found to be higher for [b2mpy][Tf_2_N] than for [b4mpy][Tf_2_N], implying stronger intermolecular forces not only at the surface but also in the bulk.^27^

Figure 3 shows that the isobaric molar heat capacity presents a linear behavior with temperature. For both ILs, this property increases with increasing temperature. The values of the isobaric molar heat capacity were close for both isomers, with a slightly higher value observed for [b2mpy][Tf_2_N] than for [b4mpy][Tf_2_N]. The isobaric molar heat capacity of a liquid refers to the quantity of energy per mole that the compound can store before its temperature increases. It appears that the methyl group at position 2 of the pyridine ring is more effective at storing energy than when it is at position 4. The results obtained are in accordance with those reported by Gómez et al.,^14^ where the heat capacity values were in the following order: position 2 > position 3 > position 4 with respect to the methyl position of the pyridine ring for the same studied ILs.

Conversely, when considering transport properties such as dynamic viscosity and electrical conductivity, a different temperature behavior was noted, which can be described with an exponential form equation such as the Vogel–Fulcher–Tammann equation:^34−36^
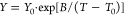
4where *Y* is η or κ and *Y*_0_, *B*, and *T*_0_ are adjustable parameters. These parameters with the corresponding absolute average relative deviations are also included in Table 3.

The variation of dynamic viscosity with temperature is presented in Figure 4. As shown, the dynamic viscosity values exhibit convex curved profiles, decreasing considerably with temperature. At lower temperatures, the values are higher for [b2mpy][Tf_2_N] than for [b4mpy][Tf_2_N]. However, these differences reduce with increasing temperature. The obtained results could be explained by two main factors: first, this property strongly depends on intermolecular interactions and structural characteristics.^6,31^ Consequently, higher viscosities are expected for [b2mpy][Tf_2_N] than for [b4mpy][Tf_2_N], as observed experimentally. Second, the presence of the methyl group in the *ortho* position in the pyridinium ring affects the steric hindrance and spatial arrangement around the nitrogen atom. This can limit the rotation of the alkyl chain around the nitrogen atom.^10^ Based on this, [b2mpy][Tf_2_N] is expected to be more viscous than its isomer, which is confirmed by our measurements.

The electrical conductivity plots as a function of temperature are depicted in Figure 5. The results indicate a considerable increase in the electrical conductivity of the examined ILs with temperature. Notably, [b4mpy][Tf_2_N] is more conductive than [b2mpy][Tf_2_N] at elevated temperatures. These differences between the two ILs become more pronounced with increasing temperature. The experimental results can be explained considering the relation between transport properties and ion mobility. Particularly, a high ion mobility within the fluid suggests lower viscosity and higher electrical conductivity.^37^ The experimental results agree with this statement.

Table 4 compares the literature values with our experimental results in terms of AARD. It should be mentioned that the values of thermophysical properties are mainly influenced by the water content and the measurement method. The first factor is particularly fundamental in the transport properties of dynamic viscosity and electrical conductivity. The measurement method is reflected above all in the measurement of densities: when determined using a vibrating tube, the results are more reliable with deviations of ∼0.03%; meanwhile, when determined using a pycnometer, the deviations are considerably greater, from 1 to 4.4%. There are only three studies reporting the thermophysical properties of 1-butyl-2-methylpyridinium bis(trifluoromethylsulfonyl), i.e., two for isobaric molar heat capacities^14,15^ and another for densities, dynamic viscosities, and surface tensions;^16^ in all cases, the deviations were moderate. For 1-butyl-4-methylpyridinium bis(trifluoromethylsulfonyl), there were substantially more studies covering all thermophysical properties presented in the present study. Regarding the deviations, there were small ones and some were somewhat larger, although none exceeded 10%. The greatest deviation occurred for isobaric molar heat capacities, with a deviation of 9.74%; for this property, there were two studies that reported deviations of only 0.44 and 1.25%.

**Table 4:** Absolute Average Relative Deviations, AARD, among Our Experimental and Literature Data

refs	*T*/K	ρ (%)	*n*_D_ (%)	σ (%)	*C*_p,m_ (%)	η (%)	κ (%)
1-Butyl-2-methylpyridinium bis(trifluoromethylsulfonyl)imide							
Gómez et al.^14^	308.15–323.15				4.10		
Zorębski et al.^15^	293.15–323.15				2.90		
Bittner et al.^16^	293.15–323.15	2.66		6.30		5.85	
1-Butyl-4-methylpyridinium bis(trifluoromethylsulfonyl)imide							
Gómez et al.^14^	308.15–323.15				0.44		
Zorębski et al.^15^	293.15–323.15				1.25		
Bittner et al.^16^	293.15–323.15	0.96		5.40		2.80	
Oliveira et al.^17^	278.15–323.15	0.03				5.60	
Zhang et al.^18^	298.15						1.20
Liu et al.^19^	278.15–323.15	0.49				1.93	3.84
Larriba et al.^20^	293.15–323.15	0.02	0.01			4.65	
Larriba et al.^21^	296.2–320.2				9.74		
Andanson et al.^22^	298.15	0.03				5.70	
Papaiconomou et al.^23^	298.15	4.40					

## 4 Conclusions

A thermophysical investigation was conducted on two pyridinium-based ILs, namely, 1-butyl-2-methylpyridinium bis(trifluoromethylsulfonyl)imide and 1-butyl-4-methylpyridinium bis(trifluoromethylsulfonyl)imide. This study was performed in the temperature range of 278.15-338.15 K and ambient pressure of 100 kPa. The experimental properties of the ILs that were examined included density, speed of sound, refractive index, surface tension, molar isobaric heat capacity, kinematic viscosity, and electrical conductivity. Additionally, derived properties such as isentropic compressibility, molar refraction, and dynamic viscosity were calculated. The objective was to analyze the influence of the substituent position in the pyridinium ring on these thermophysical properties of the ILs.

## Supporting Information Available

Plots for density, speed of sound, refractive index, and surface tension as a function of temperature (PDF)

## Abbreviations

density: ρ
speed of sound: *c*
refractive index: *n*_D_
surface tension: σ
as a function of temperature, *T*: at *p* = 100 kPa of the studied ionic liquids.
