# Supplementary material for: Thermophysical Study of 1-Butyl-2-methylpyridinium bis(trifluoromethylsulfonyl)imide and 1-Butyl-4-methylpyridinium bis(trifluoromethylsulfonyl)imide
Source: J Chem Eng Data. 2024 Sep 10;69(11):3739–46. doi: 10.1021/acs.jced.4c00255 (PMC11573118; doi:10.1021/acs.jced.4c00255)
Supplement: Supplementary file 1 — je4c00255_si_001.pdf [file je4c00255_si_001.pdf]

SUPPORTING INFORMATION

Thermophysical Study of 1-Butyl-2-methylpyridinium bis (trifluoromethylsulfonyl) imide and 1-Butyl-4-methylpyridinium bis (trifluoromethylsulfonyl) imide

Hamid Djebouri [a], Saeda Didaoui [a], Sahar Mrad [b], Alberto Lafuente [c], and Carlos Lafuente [c,*]

[a] Laboratory of Thermodynamics and Molecular Modeling, Faculty of Chemistry, University of Sciences and Technology Houari Boumediene, BP 32, El Alia,Algiers, 16111, Algeria

[b] Université de Tunis El Manar, Faculté des Sciences, Laboratoire des Matériaux, Cristallochimie et Thermodynamique Appliquée, LR15ES01, Département de Chimie, 2092 Tunis, Tunisia

[c] Departamento de Química Física, Facultad de Ciencias, Universidad de Zaragoza, 50009, Zaragoza, Spain

*Corresponding author. E-mail address: celadi@unizar.es

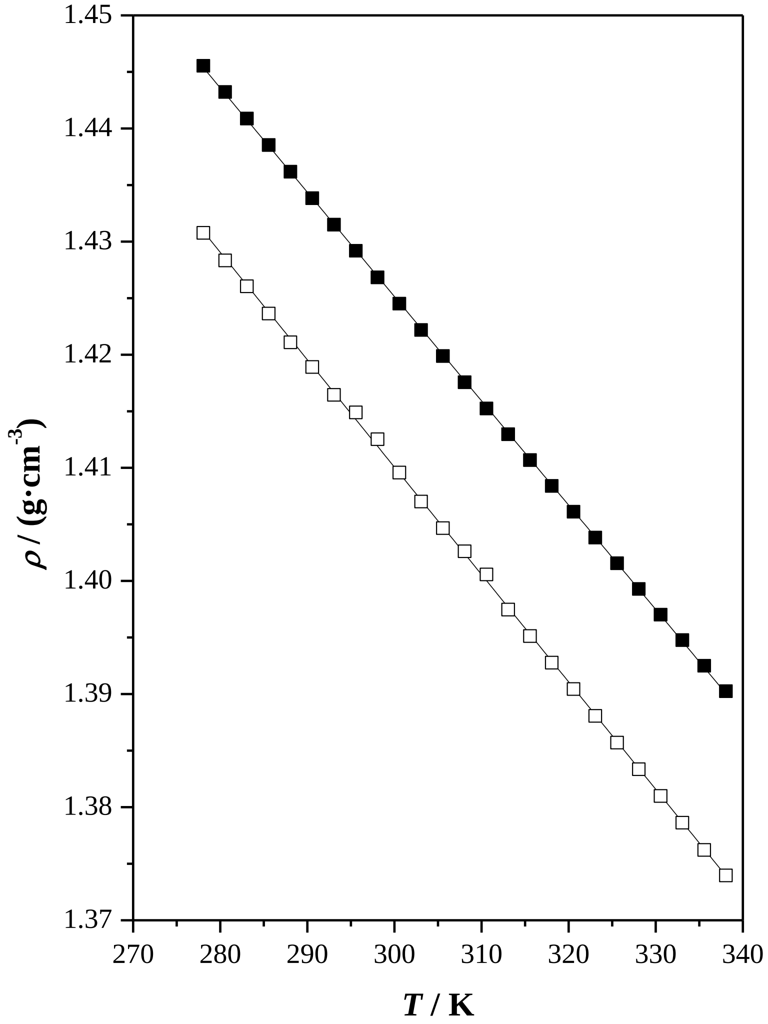

Figure S1. Density, ρ, as a function of temperature, T, at p = 100 kPa of the studied ionic liquids: [b2mpy][Tf_2N] (■); [b4mpy][Tf_2N] (□); (—) correlated values.

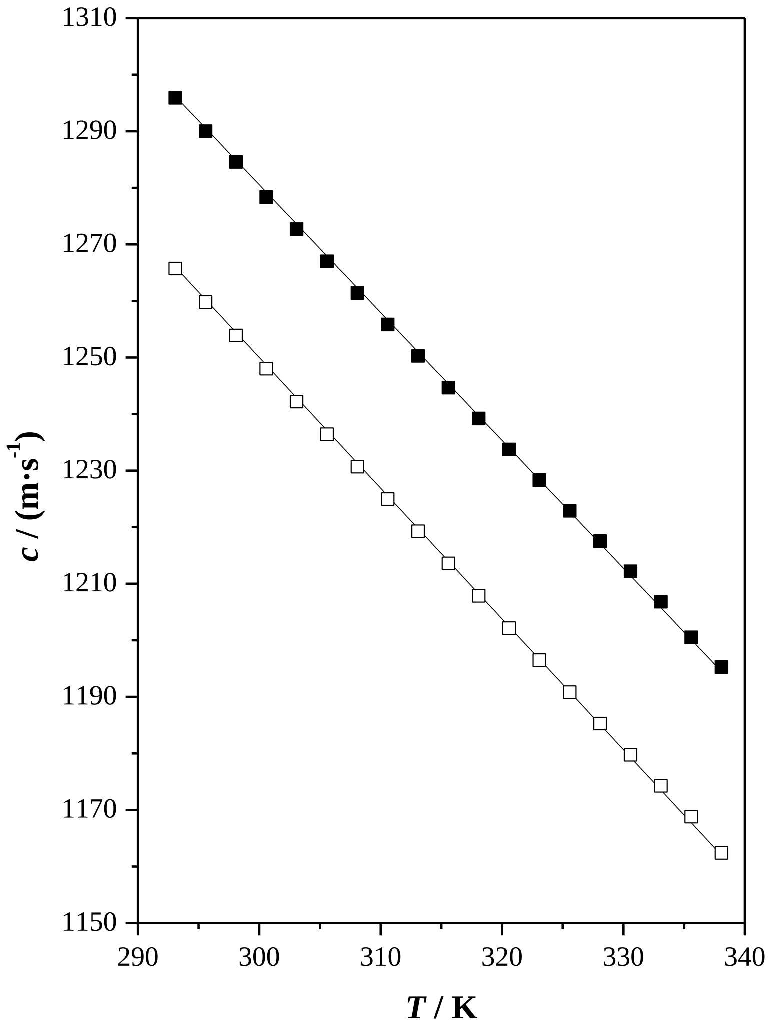

Figure S2. Speed of sound, *c*, as a function of temperature, *T*, at p = 100 kPa of the studied ionic liquids: [b2mpy][Tf_2N] (■); [b4mpy][Tf_2N] (□); (—) correlated values.

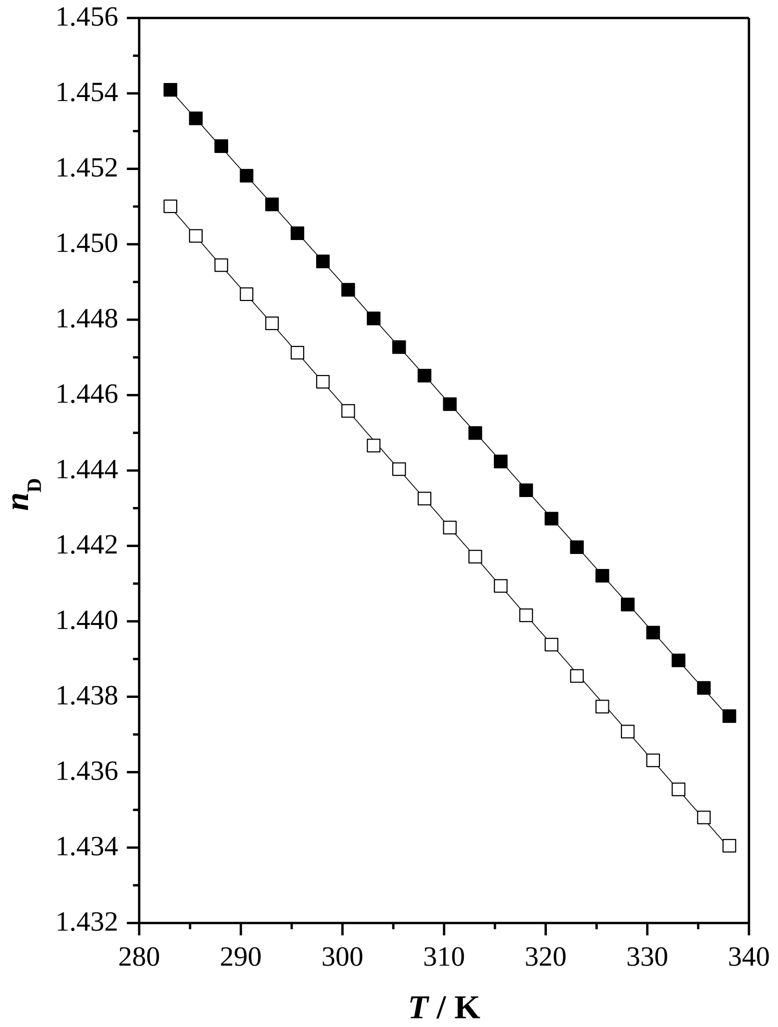

Figure S3. Refractive index, n_D, as a function of temperature, *T*, at p = 100 kPa of the studied ionic liquids: [b2mpy][Tf_2N] (■); [b4mpy][Tf_2N] (□); (—) correlated values.

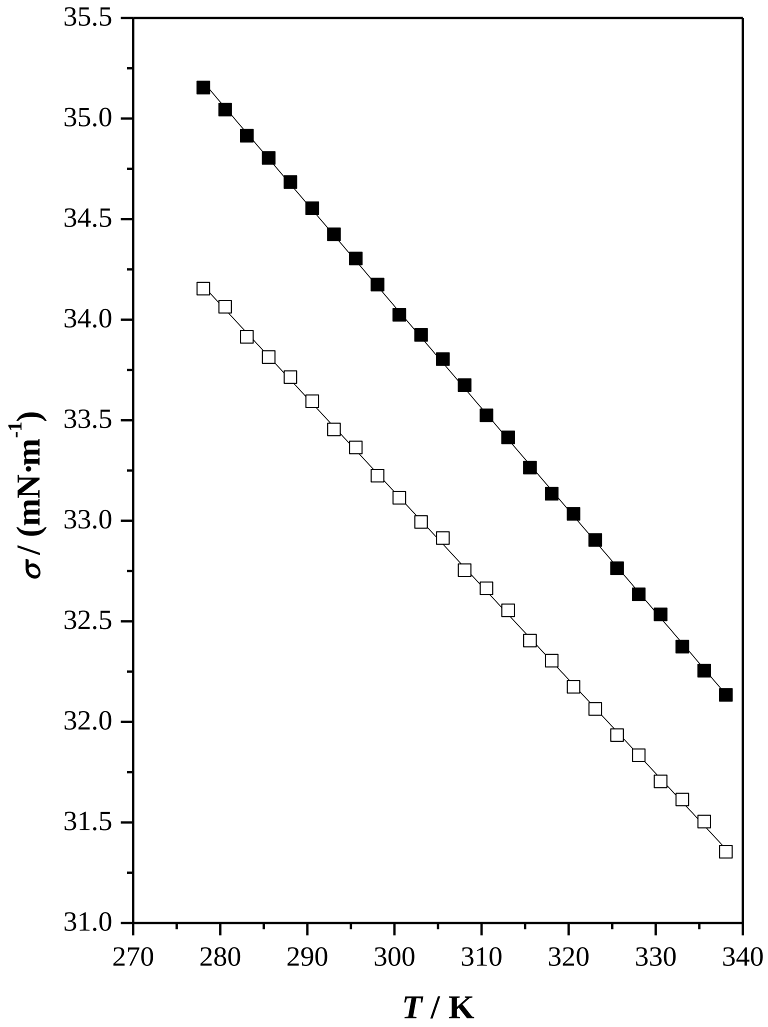

Figure S4. Surface tension, *σ*, as a function of temperature, *T*, at *p* = 100 kPa of the studied ionic liquids: [b2mpy][Tf_2N] (■); [b4mpy][Tf_2N] (□); (—) correlated values.
